# Mini-midvastus versus medial parapatellar approach in total knee arthroplasty: difference in patient-reported outcomes measured with the Forgotten Joint Score

**DOI:** 10.1186/s13018-020-01869-2

**Published:** 2020-08-17

**Authors:** Wei Lin, Jinghui Niu, Yike Dai, Guangmin Yang, Ming Li, Fei Wang

**Affiliations:** Department of Orthopedic Surgery, Third Hospital of Hebei Medical University, No. 139 Ziqiang Road, Shijiazhuang, 050051 Hebei People’s Republic of China

**Keywords:** Mini-midvastus approach, Medial parapatellar approach, Total knee arthroplasty, Forgotten Joint Score

## Abstract

**Background:**

Low knee awareness after minimally invasive total knee arthroplasty (TKA) has become the ultimate target of a natural-feeling knee that meets patient expectations. The objective of this research was to compare the clinical outcomes of TKA via the mini-midvastus (MMV) approach or the medial parapatellar (MPP) approach, and to evaluate which approach can lead to a better quality of life after surgery.

**Methods:**

From January 2015 to December 2016, a retrospective cohort study was conducted in 330 patients who underwent TKA via a mini-midvastus (MMV) approach. During this period, we also selected 330 patients who underwent TKA via a medial parapatellar (MPP) approach (MPP group) for comparison. Clinical results were assessed with the visual analog scale score for pain, range of motion, and the Knee Society Score. The Forgotten Joint Score was used to analyze the ability to forget the joint.

**Results:**

There were significant differences with regard to visual analog scale score, range of motion, and the Knee Society Score until 6 months after surgery between the MMV and MPP groups (*p* < 0.05), but the differences were not significant at 12 months, 24 months, and 36 months after surgery. However, there were significant differences in the Forgotten Joint Score between the groups during the follow-up period (*p* < 0.05).

**Conclusion:**

When forgetting the artificial joint after TKA is the ultimate target, better quality of life can be acquired by performing TKA via the MMV approach. In addition, compared with the MPP approach, the MMV approach can offer less pain and a faster recovery.

## Background

Total knee arthroplasty (TKA) is the best choice for the treatment of end-stage osteoarthritis, and it can restore knee function, relieve pain, and improve quality of life (QOL), with 95% of patients achieving a good prosthesis survival rate [1, 2]. There are several surgical approaches for primary TKA, but it remains controversial which approach can achieve the best postoperative results.

Although the medial parapatellar (MPP) approach affords excellent surgical visualization [3], it injures the quadriceps tendon and may cause weakened extensor function, and thus, the functional outcome remains unsatisfactory [4]. To the contrary, the mini-midvastus (MMV) approach not only reduces the injury to the quadriceps, but is also associated with improved postoperative outcomes [5]. Simultaneously, the MMV approach has also been popularized, and compared with TKA via the standard approach, it has led to earlier postoperative flexion and a higher mean Knee Society Score (KSS) [6–8].

The traditional evaluation system often focuses on the objective evaluation of surgeons when evaluating the postoperative results of TKA. However, the concerns of patients after TKA are not always consistent with the surgeon’s assessment [9, 10]. Therefore, there is a growing tendency to use patient-reported outcome measure (PROM) tools to evaluate patient outcomes [11].

The Forgotten Joint Score (FJS) is a PROM tool designed to assess patients’ ability to lose joint awareness and awareness that their joint is artificial [12]. The ability of forgetting an artificial joint has been seen as the ultimate goal of joint replacement and can reflect patient satisfaction [12, 13]. So far, few studies have evaluated the change in joint awareness after TKA via different approaches. It is important to clearly understand the actual changes in joint awareness after TKA via different approaches.

The objective of this research was to conduct a retrospective cohort study to investigate the functional outcomes of TKA via the MMV or MPP approaches using the FJS, and evaluate which approach can lead to better QOL after surgery.

## Methods

After approval from the Institutional Review Committee, we performed a retrospective cohort study from January 2015 to December 2016. We included 330 patients who underwent primary TKA via the MMV approach in the MMV group. To improve the reliability of this research, we used a 1:1 ratio with regard to age, sex, body mass index (BMI), and follow-up time to select 330 patients who underwent primary TKA via the MPP approach (MPP group) for comparison.

Our eligibility criteria were (1) unilateral primary knee osteoarthritis and Kellgren and Lawrence [14] grade ≥ 3 of the tibiofemoral joint, (2) primary cruciate-retaining TKA, (3) flexion-contracture deformity < 20°, and (4) varus deformity < 20° [15]. Patients who had valgus or stiff knees, neurological problems, revision TKA, or previous open knee surgery were excluded.

### Surgical procedures

All patients received the same anesthesia, and all surgeries were performed in our center by the same senior orthopedic surgeon. In both groups, as described by Liu et al. [16], all surgeries were performed through a midline skin incision. Using standard surgical instruments, extramedullary alignment for the tibial component and intramedullary alignment for the femoral component were obtained. In the MMV group, we dissected the vastus medialis obliquus at a distance of no more than 3 cm from the superior pole of the patella. In the MPP group, we performed superior extension of no more than 3 cm into the quadriceps tendon. The patella was subluxated laterally without eversion, and soft tissue balance was achieved in a standard method. The patellar prostheses were not replaced, and the patellar surface was only reshaped to fit the prosthesis. In all patients, we used the same cemented knee prosthesis (cruciate-retaining, LINK, Hamburg, Germany, Gemini MK II). After the total knee prosthesis was implanted, the wound was closed in a standard layered fashion.

### Postoperative treatment

All patients received the same postoperative pain control and rehabilitation programs [17]. After surgery, the patients were asked to walk with load-bearing as soon as possible. Functional exercises and physical therapy were best started on the first day, and active and passive extension and flexion exercises of the knee were performed for at least 3 months.

### Outcome measures

Assessments were performed by a senior orthopedic surgeon who did not attend the treatments. Parameters including operative time, tourniquet time, skin incision length, and time to straight leg raise for all patients were recorded after surgery.

The visual analog scale (VAS) score for pain, range of motion (ROM), and Knee Society Score (KSS) [18] were assessed. For comparing the postoperative status of the patients who received TKA via the two different approaches, we used the Forgotten Joint Score (FJS; a 12-item questionnaire with a maximum score of 100) to analyze the ability to forget the joint [12]. Higher scores represented better results. All data were assessed at 1 month, 6 months, 12 months, 24 months, and 36 months after surgery.

Standard anteroposterior and lateral radiographs were used for all preoperative and postoperative radiologic evaluations. Component and overall alignment of neutral ± 3° was rated as satisfactory.

### Statistical analysis

The normality of the continuous variables was checked with the Shapiro-Wilk test. If the data were normally distributed, the variables were checked with Student’s *t* test; if not, a non-parametric test was selected. Categorical variables were checked with Fisher’s exact test or chi-square test. The correlations between the FJS and surgical approach (MMV versus MPP), sex, gender, and BMI were analyzed by multiple linear regression. The data were analyzed with SPSS 23.0 (IBM, Chicago, IL, USA). A *p* < 0.05 was considered statistically significant.

## Results

All patients were followed up for at least 3 years. No significant differences were found for demographic parameters between the MMV group and the MPP group (*p* > 0.05) (Table 1). We found dramatic differences in skin incision length (9.4 ± 3.2 versus 12.8 ± 2.6; *p* < 0.05) and straight leg raise (1.3 ± 0.7 versus 2.8 ± 0.6; *p* < 0.05) between the two groups (Table 2).

**Table 1:** Patients’ demographics in the two groups

Demographics	MMV group	MPP group	*p* value
Total patients	330	330	–
Age (years)	65.2 ± 7.7	66 ± 8.1	0.08
BMI (kg/m^2^)	26.2 ± 3.9	25.6 ± 3.7	0.46
Sex			0.58
Male	82 (24.8%)	76 (23%)	–
Female	248 (75.2%)	254 (77%)	–
Follow-up time (years)	3.5 ± 0.4	3.6 ± 0.3	0.51

*MMV* mini-midvastus, *MPP* medial parapatellar, *BMI* body mass index; mean ± standard deviation

**Table 2:** Postoperative clinical results in the two groups

Results	MMV group	MPP group	*p* value
Operative time (min)	83.1 ± 8.4	81.8 ± 7.2	0.382
Tourniquet time (min)	41.1 ± 4.2	39.2 ± 3.4	0.421
Skin incision length (cm)	9.4 ± 3.2	12.8 ± 2.6	0.032
Straight leg raise (day)	1.3 ± 0.7	2.8 ± 0.6	0.026

*MMV* mini-midvastus, *MPP* medial parapatellar; mean ± standard deviation

The VAS, ROM, and KSS in the MMV group were better than those in the MPP group within 6 months (*p* < 0.05), but no significant differences were found at 12 months, 24 months, and 36 months after surgery (*p* > 0.05) (Tables 3 and 4). However, during follow-up, the FJS in the MMV group was higher than in the MPP group (*p* < 0.05) (Table 5). The multiple linear regression showed that a higher FJS was correlated with the MMV approach (*p* < 0.05) (Table 6).

**Table 3:** The VAS and ROM in the two groups

	MMV group	MPP group	*p* value
VAS			
Preop	5.1 ± 0.7	5.3 ± 0.8	0.421
Postop 1 month	3.4 ± 0.7	4.5 ± 1.1	0.032
Postop 6 months	3.1 ± 0.8	4.1 ± 0.9	0.043
Postop 12 months	2.8 ± 1.2	2.9 ± 1.1	0.072
Postop 24 months	2.3 ± 0.8	2.4 ± 0.7	0.771
Postop 36 months	2.1 ± .7	2.2 ± 1.1	0.881
ROM			
Preop	97.8 ± 8.9	97.1 ± 6.7	0.615
Postop 1 month	103.9 ± 7.4	98.6 ± 7.3	0.034
Postop 6 months	105.1 ± 5.4	100.8 ± 7.7	0.041
Postop 12 months	106.3 ± 7.8	104.1 ± 8.2	0.064
Postop 24 months	109.9 ± 6.8	108.6 ± 7.3	0.525
Postop 36 months	112.9 ± 7.8	110.2 ± 7.2	0.846

*MMV* mini-midvastus, *MPP* medial parapatellar, *VAS* visual analog score for pain; *ROM* range of motion; *Preop* preoperation, *Postop* postoperation; mean ± standard deviation

**Table 4:** The KSS in the two groups

	MMV group	MPP group	*p* value
Clinical score			
Preop	36.5 ± 4.8	36.7 ± 5.4	0.681
Postop 1 month	71.6 ± 5.7	68.2 ± 6.5	0.032
Postop 6 months	76.3 ± 4.9	73.6 ± 6.2	0.037
Postop 12 months	81.6 ± 5.9	80.4 ± 5.7	0.087
Postop 24 months	89.6 ± 3.2	88.4 ± 3.9	0.661
Postop 36 months	93.3 ± 4.1	92.2 ± 4.8	0.783
Functional score			
Preop	38.4 ± 3.9	37.2 ± 5.4	0.783
Postop 1 month	65.1 ± 5.9	61.2 ± 6.1	0.022
Postop 6 months	71.4 ± 4.8	67.1 ± 5.2	0.033
Postop 12 months	74.1 ± 3.1	73.4 ± 4.7	0.061
Postop 24 months	82.2 ± 3.6	81.6 ± 3.3	0.511
Postop 36 months	85.1 ± 3.7	84.1 ± 3.2	0.685

*MMV* mini-midvastus, *MPP* medial parapatellar, *KSS* Knee Society Score, *Preop* preoperation, *Postop* postoperation; mean ± standard deviation

**Table 5:** The FJS in the two groups

	MMV group	MPP group	*p* value
Postop 1 month	57.6 ± 6.9	50.4 ± 5.4	0.027
Postop 6 months	62.4 ± 7.1	55.6 ± 5.5	0.022
Postop 12 months	71.6 ± 5.1	65.3 ± 4.8	0.041
Postop 24 months	78.6 ± 6.3	70.4 ± 6.1	0.037
Postop 36 months	81.1 ± 4.1	78.2 ± 4.4	0.046

*MMV* mini-midvastus, *MPP* medial parapatellar, *FJS* Forgotten Joint Score, *Preop* preoperation, *Postop* postoperation; mean ± standard deviation

**Table 6:** Multiple linear regression analysis

	Coefficient	95% CI	*p* value
MMV approach	42.3	28.4 to 72.5	0.037
MPP approach	32.5	20.4 to 53.6	0.463
Age	0.903	0.128 to 1.431	0.537
BMI	− 0.701	− 1.814 to − 0.831	0.974
Sex	0.857	− 1.934 to 4.547	0.541

*MMV* mini-midvastus, *MPP* medial parapatellar, *BMI* body mass index, *CI* confidence interval

According to the radiographic evaluation, there were no cases of improper implant positioning in the two groups. Until the last follow-up, no significant postoperative complications were found in the patients.

## Discussion

The most important finding in our research was that when forgetting the artificial joint after TKA is the ultimate target, better QOL can be acquired by performing TKA via a MMV approach. In addition, compared with the MPP approach, the MMV approach may offer less pain and a faster recovery.

There are multiple approaches to minimally invasive TKA, including the MMV approach, the quadriceps-sparing approach, the mini-subvastus approach, and the limited MPP approach [19]. However, because of the difficulty of the operation, the long learning curve [7], and the difficulty of preserving the extensor mechanism [15], few surgeons currently use the quadriceps-sparing approach and the mini-subvastus approach. Thus, we used FJS to study whether the MMV approach can be successfully used routinely as a minimally invasive TKA approach.

Several authors have attempted to compare the MMV approach and the MPP approach with conventional scores, such as the VAS, the HSS (Hospital for Special Surgery) score, and the KSS, and only found differences in short-term outcomes, but this early clinical advantage has seemed to disappear over time [5–7]. Some authors even found no differences in clinical outcomes during the follow-up period [20–22]. A more responsive joint-specific score, such as the FJS, can provide a clearer assessment of patients’ postoperative satisfaction, and our study highlighted differences for the first time between the two approaches during a follow-up of at least 3 years. This shows that the FJS is an appropriate tool to evaluate patients’ satisfaction, which can reflect patients’ satisfaction well not only in the early postoperative period but also in the medium-term postoperative period when KSS cannot detect differences.

The FJS has been a highly rated scoring method over the last few years and is often used to measure the ability of patients to forget joint awareness or joint arthroplasty [12]. Even if the patient’s knee function is improved and no pain is felt, the FJS will be lower if the patient is “aware of” the presence of their artificial joint in daily life. As a result, minor complaints that are not identified by specific questions (such as “can you participate in sports?”) are called “aware” joints, which may more sensitively reflect postoperative patient satisfaction and reduce the ceiling effect [12, 23]. Ozaki et al. believed that the FJS is a scoring system that can express “sense of stability” as “awareness” [24]. Thomsen et al. believed that the FJS combines factors such as stiffness, pain, ability of daily activities, and patients’ expectations to reflect patients’ ability to forget artificial joints during activity, and therefore, this scoring system may be the best tool to evaluate the results after TKA [25]. Another study found that when using the FJS scoring system to evaluate the difference in knee awareness of patients who underwent patellofemoral arthroplasty, unicompartmental knee arthroplasty, and TKA, they found that patients who underwent different joint arthroplasties had large differences in the FJS [13].

Hiyama et al. found that quadriceps strength and pain were the main factors affecting joint awareness after TKA [26]. Quadriceps weakness is the main obstacle to patients’ functional recovery after TKA, and pain is usually one of the main criteria for success or failure after TKA. Quadriceps weakness and pain are closely related to disability [27], patient satisfaction [28], and QOL [29, 30].

One of the greatest advantages of TKA via the MMV approach is that it retains the extensor mechanism as much as possible during the operation. Therefore, it can reduce the perioperative pain and help the patients recover quickly. However, it has been pointed out that the standard MPP approach may decrease the strength of the quadriceps measured by isokinetics as much as 30.7% in the 2 years after TKA, and excessive damage to the extensor mechanism may be permanent [31]. Retaining the extensor mechanism as much as possible was the main reason why the MMV approach enabled patients to achieve faster functional recovery and higher satisfaction [8, 32]. Our study adds to these findings by investigating the effects of quadriceps weakness and pain on joint awareness after TKA.

Some studies indicated that the performance of a postoperative straight leg raise reflects the recovery of quadriceps muscle strength [33, 34]. Schroer and Nestor measured the pre- and postoperative muscle strength of their patients who underwent TKA via the MMV approach and reported that patients had regained their preoperative quadriceps muscle strength in a short period, and even exceeded those levels by 30% at 3 to 6 months [35, 36]. Similar results were found in our research. In the present study, the time to be able to perform a postoperative straight leg raise in patients who underwent TKA via the MMV approach was much earlier than in patients who underwent TKA via the MPP approach. This difference in quadriceps muscle strength is essential for patients to resume daily activities. As reported in previous studies [37, 38], we also found that the MMV approach can shorten the length of the skin incision compared with the traditional MPP approach. In addition, a shorter skin incision may produce a better esthetic effect, which can improve patients’ satisfaction.

However, it has been pointed out that during the TKA with minimally invasive surgery, such as the MMV approach, the complex manipulation and poor exposure can lead to malalignment of the components [34, 39], which may lead to failure of TKA [40]. However, in our study, no significant differences in postoperative complications were found in the patients until the final follow-up. Consequently, the MMV approach that protected the extensor mechanism may be a good choice in TKA.

The limitation of this study was that it had a retrospective mid-term follow-up design. Prospective and longer-term studies should be performed to confirm these findings. In addition, further study is needed to determine the minimal clinically important difference (MCID) threshold of FKS between the two groups.

## Conclusion

When forgetting the artificial joint after TKA is the ultimate target, better quality of life can be achieved by performing a TKA via the MMV approach. In addition, compared with the MPP approach, the MMV approach may offer less postoperative pain and a faster recovery.

## Data Availability

The detailed data and materials of this study are available from the corresponding author via e-mail on reasonable request.

## Abbreviations

TKA: Total knee arthroplasty
MMV: Mini-midvastus
MPP: Medial parapatellar
PROM: Patient-reported outcome measure
QOL: Quality of life
ROM: Range of motion
KSS: Knee Society Score
FJS: Forgotten Joint Score
BMI: Body mass index
